# The Longitudinal Aging Study Amsterdam: design and cohort update 2025

**DOI:** 10.1007/s10654-025-01238-5

**Published:** 2025-05-14

**Authors:** Emiel O. Hoogendijk, Natasja M. van Schoor, Yuwei Qi, Marjolein Visser, Joukje C. Swinkels, Marjolein I. Broese van Groenou, Almar A. L. Kok, Tjalling J. Holwerda, H. Roeline W. Pasman, Bregje D. Onwuteaka-Philipsen, Sharon Remmelzwaal, Erik van Ingen, Theo G. van Tilburg, Aimée-Claire van Haaster, Marleen van der Horst, Jan Poppelaars, Dorly J. H. Deeg, Martijn Huisman

**Affiliations:** 1 Department of Epidemiology & Data Science, Amsterdam Public Health Research Institute, Amsterdam UMC– Location VU University Medical Center, Amsterdam, The Netherlands; 2 Department of General Practice, Amsterdam Public Health Research Institute, Amsterdam UMC– Location VU University Medical Center, Amsterdam, The Netherlands; 3 Department of Health Sciences, Faculty of Science, Amsterdam Public Health Research Institute, Vrije Universiteit Amsterdam, Amsterdam, The Netherlands; 4 Department of Sociology, Faculty of Social Sciences, Vrije Universiteit Amsterdam, Amsterdam, The Netherlands; 5 Department of Psychiatry, Amsterdam Public Health Research Institute, Amsterdam UMC– Location VU University Medical Center, Amsterdam, The Netherlands; 6 Department of Public and Occupational Health, Amsterdam Public Health Research Institute, Amsterdam UMC– Location VU University Medical Center, Amsterdam, The Netherlands

**Keywords:** Longitudinal studies, Netherlands, Epidemiology, Aging, Study design, Cohort studies, Mental health, Loneliness, Chronic disease, COVID-19, Frailty, Intrinsic capacity

## Abstract

The Longitudinal Aging Study Amsterdam (LASA) is an ongoing prospective cohort study of older adults in the Netherlands, with data on multiple domains of functioning available over a period of more than 30 years of follow-up. The study started in 1992 with a nationally representative sample of older adults aged 55–84 years. Over the years, three refresher cohorts (two cohorts aged 55–64 years in 2002 and in 2012, and one cohort aged 60–86 years in 2024) were added. The main aim of LASA was to describe determinants, trajectories and consequences of (changes in) physical, cognitive, emotional and social functioning. LASA has multiple strengths, including its multidisciplinary character, the very long period of follow-up, and the cohort-sequential design which enables the study of longitudinal changes as well as historical time trends in functioning. So far, findings based on data from LASA have been reported in more than 800 scientific publications (see www.lasa-vu.nl). In this article, we provide an update of the design and methods of LASA, including a description of several ancillary studies such as the Loneliness study and the COVID-19 study.

## Introduction

The Longitudinal Aging Study Amsterdam (LASA) is an ongoing prospective cohort study among older adults in the Netherlands. The study started in 1992, initiated by the Dutch Ministry of Welfare, Health and Culture (currently Ministry of Health, Welfare and Sport). The idea for the study was developed at a time when the aging of the Dutch population was becoming a concrete prospect for policymakers in the field of care for older adults. The underlying vision was that high-quality empirical data was needed to inform policy and that these data should reflect an interdisciplinary approach, with contributions from medical and social scientists.

This interdisciplinary approach was reflected in the original aim and research questions of LASA. The main objective of LASA was to study the determinants, trajectories and consequences of (changes in) physical, cognitive, emotional and social functioning [1–3]. Overarching research questions were: (1) Which changes over time take place in the physical, cognitive, emotional and social domains of functioning in older persons? (2) What are predictors of change of these domains of functioning? (3) How are changes in these domains of functioning interrelated? (4) What are the consequences of changes in functioning for older adults’ contributions to society, the necessity of adjustment, and the need for care? As the study progressed, additional research questions have been formulated that guided ancillary data collections and research. Many of these developments have been described in the original cohort profile and previous cohort updates of LASA [1–3].

LASA is still one of the few longitudinal aging studies in the Netherlands [4, 5], and worldwide one of the few with data over a period of more than 30 years. Other strengths of the study include its multidisciplinary character and its cohort-sequential design which enables the study of longitudinal trajectories, cohort differences and time trends in various domains of functioning [6–8].

Since the publication of the 2019 cohort update of LASA [3], several new data collections have been completed. In this paper, we briefly describe the design of LASA and provide an update of the methods, including a description of the design of several ancillary studies that have been conducted in the past few years such as the COVID-19 study, the Loneliness study and the Family caregiving study.

## The design of LASA

The LASA study started in 1992, with a cohort based on a representative sample of older people aged 55–84 years (born between 1908 and 1937) from three regions in the Netherlands. The three regions (in and around the cities of Zwolle, Oss, and Amsterdam) were chosen to obtain an approximate representation of the Dutch older population, covering different cultures, religions and population densities. The three regions cover the predominantly protestant northeast, the largely catholic south and the more secularized western part of the Netherlands, and include both urbanized and rural areas. The initial LASA sample was derived from respondents of the NESTOR (Netherlands Program for Research on Aging) study on Living arrangements and Social Networks of older adults (LSN) in the Netherlands [9]. The LSN study was based on a sample randomly selected from the registers of municipalities in the three regions in 1992, with an oversampling of the oldest old and men, to ensure that there would be reasonable numbers of respondents after several years of follow-up. The initial sample of the LSN study consisted of 3805 older people, which corresponds to a response rate (defined as the number of complete and partial interviews, divided by the total number of eligible persons in the sample plus a fraction of those persons who were in the sample but of whom eligibility could not be determined) of 60%. The cooperation rate (defined as the number of completed interviews divided by the total number of contacted eligible persons) was 62%.

**Fig. 1:**
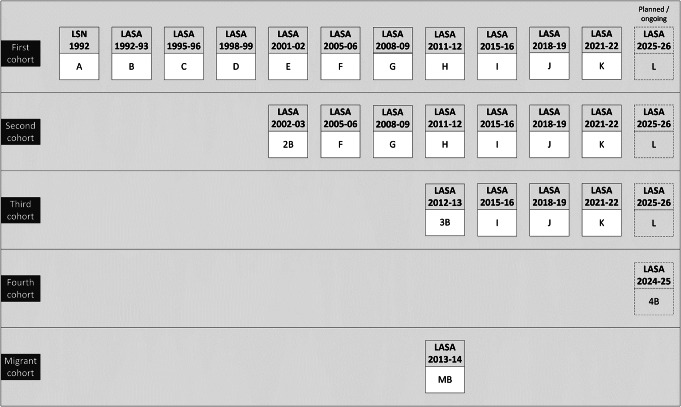
Cohorts and measurement waves of LASA

**Fig. 2:**
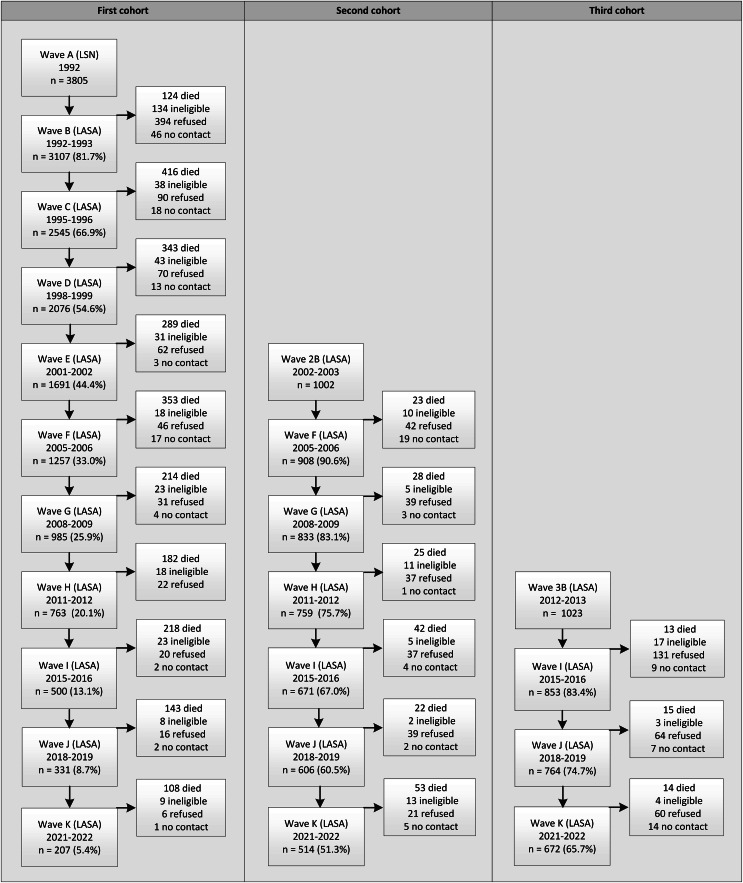
Flowchart of the main measurement waves of the LASA study

On average 11 months after the LSN interview (wave A), respondents were invited to participate in the first measurement wave of LASA (wave B, *n* = 3107). The response rate of this first LASA wave was 85% and the cooperation rate was 89%. Since the 1992 LSN measurement wave, there have been 10 main LASA measurement waves to date (Figs. 1 and 2). At the tenth measurement wave in 2021–2022 (wave K), approximately 29 years after the start of the study, a total of 207 respondents from the original cohort continued to participate.

To be able to study cohort differences and to keep the LASA study sample sufficiently large, two refresher cohorts of people aged 55–64 years were added to the original sample in 2002–2003 and 2012–2013 from the same sampling frames, exactly 10 and 20 years after the start of LASA. Figure 2; Table 1 show the sample sizes for all the cohorts across all measurement waves and by interview type. The second cohort (wave 2B, included in 2002–2003) consisted of 1002 men and women born between 1938 and 1947 (cooperation rate = 62%), and the third cohort (wave 3B, included in 2012–2013) consisted of 1023 men and women born between 1948 and 1957 (cooperation rate = 63%). In follow-up measurements, respondents from these new cohorts were merged with those from the original cohort. In 2024, a fourth cohort (wave 4B) was added consisting of a sample from the same sampling frame of men and women born between 1938 and 1964, aged 60–86 years (data collection still ongoing). Subsequently, in 2025 the data collection of a new regular measurement wave will start (wave L), consisting of all remaining respondents from the first three cohorts.

**Table 1:** LASA interview type by measurement wave

Wave	Year	Cohort^a^	Age range	*N*	*n* by interview type		
					Face-to-face	Telephone	
					General interview (medical interview)	Respondent	Proxy
B	1992–1993	Cohort 1	55–84	3107	3107 (2671)	-	-
C	1995–1996	Cohort 1	57–88	2545	2302 (1509)^b^	164	79
D	1998–1999	Cohort 1	60–91	2076	1874 (1382)^c^	127	75
E	2001–2002	Cohort 1	63–94	1691	1474 (1307)	122	95
2B	2002–2003	Cohort 2	55–64	1002	1002 (919)	-	-
F	2005–2006	Cohort 1, 2	57–98	2165	1908 (1805)	140	117
G	2008–2009	Cohort 1, 2	60–100	1818	1601 (1494)	117	100
H	2011–2012	Cohort 1, 2	63–104	1522	1308 (1212)	99	115
3B	2012–2013	Cohort 3	55–64	1023	1023 (889)	-	-
MB	2013–2014	Migrant cohort	55–64	478	478 (344)	-	-
I	2015–2016	Cohort 1, 2, 3	57–102	2024	1770 (1642)	148	106
J	2018–2019	Cohort 1, 2, 3	61–101	1701	1393 (1328)	198	110
K	2021–2022	Cohort 1, 2, 3	63–100	1393	1110 (1050)	195	88

^a^Birth years Cohort 1: 1908–1937, Cohort 2: 1938–1947, Cohort 3: 1948–1957, Migrant cohort: 1948–1957

^b^In 1995–1996, only people born before 1931 were asked to participate in the medical interview

^c^In 1998–1999, only people born before 1931 plus a control group of remaining birth years were asked to participate in the medical interview

The LASA study is conducted in line with the Declaration of Helsinki and received approval by the medical ethics committee of the VU University medical center in Amsterdam (IRB numbers: 92/138, 2002/141, 2012/361, and 2016.301).

## Data collection

Data are collected by trained interviewers who visit the respondents every 3 or 4 years at home. All interviews are audio-recorded (after consent of the respondent) for quality checks. The data collection of LASA involves both questionnaires and clinical tests, and includes measures for each of the four domains of functioning: physical, cognitive, emotional, and social. The main predictors and outcome measures in LASA have been published before [2]. Detailed descriptions of the measurements and procedures can be found on the LASA website (www.lasa-vu.nl). For purposes of longitudinal comparison, most measures of LASA remain the same across measurement waves. However, sometimes measurement instruments are updated, novel measurements are added or outdated ones are removed. In the past 5 years, various new measurements were added, such as existential loneliness (wave J), coffee and tea consumption (wave J and K), generativity (wave J and K), gender identity (wave J and K), and health literacy (wave K).

Each LASA measurement wave has three elements: a general computer-assisted personal interview, a self-administered questionnaire, and a medical computer-assisted personal interview. The general interview takes, on average, almost 2 h to complete. An abbreviated interview is offered to respondents for whom a full interview is too burdensome to complete. During the home visit for the general interview, respondents are asked to fill out a self-administered questionnaire, which is left at the respondent`s home in print, or can be accessed online (as of 2015). Respondents have three options to hand in this questionnaire: during the medical interview (which takes place approximately 4 to 6 weeks after the general interview), by postal mail, or online. At the end of the general interview, respondents are invited to participate in a subsequent medical interview. After consent, a separate visit is scheduled for this. The medical interview contains various additional questionnaires and clinical measurements, and takes on average 1 h and a half to complete. Respondents who scored highly on the symptoms checklist of depression or anxiety during the general interview are invited for an additional diagnostic psychiatric interview (Composite International Diagnostic Interview, CIDI).

As of the first follow-up wave of each cohort, a telephone interview (or proxy telephone interview) is offered to respondents who refused or were not able to participate in a full or an abbreviated face-to-face interview. The telephone interview takes approximately 15 min and includes a selection of measures from various domains of functioning. More information on the content of the telephone interviews has been published previously [3]. Over the years, the proportion of telephone interviews (respondent and proxy) has been increasing from around 10% in 1995–1996 (wave C) to around 20% in 2021–2022 (wave K). Additional analysis shows that the proportion of respondents from cohort 3 that has a telephone interview is higher than in cohorts 1 and 2. To minimize selection bias, users of LASA data are recommended to use telephone data in addition to face-to-face data, whenever the research question and available data allow it.

At waves B, C, 2B, G and 3B, blood samples were obtained from respondents who participated in the medical interview. The biomaterial measurements that were done among LASA respondents, including DNA extraction, have been described previously [2, 3]. Data on vital status of respondents is retrieved approximately every 2 years from registers of municipalities where respondents are living (currently available up to September 2023). In addition, updated information on causes of death is obtained from Statistics Netherlands, which is now available for respondents deceased until 2021.

## Attrition and representativeness

Longitudinal studies on aging are bound to incur substantial attrition of respondents over time. The main reason for drop-out in LASA is mortality [1–3, 10]. To examine the representativeness of the sample we conducted analyses in which we compared sex, year and age-stratified mortality among LASA respondents with mortality in the Dutch general population obtained from Statistics Netherlands [11]. For the analyses in this cohort update, we added data up to and including 2020. Although mortality was slightly higher in the general population compared to the LASA sample, for most groups the differences did not exceed 1% point (Table 2). This means that mortality in the LASA sample is not substantially different from mortality rates in the Dutch general older population.

**Table 2:** Mortality among LASA respondents compared to the Dutch general population

	Weighted sum of difference (LASA minus Dutch general population)^a,b,c^	
	Men	Women
Total	-0.65	-0.48
By year		
1994	-0.03	-0.19
1997	-0.59	-0.55
2000	-1.04	-0.78
2004	-0.77	-0.55
2007	-0.78	-0.27
2010	-1.08	-0.50
2014	-0.54	-0.37
2017	-0.72	-0.61
2020	-0.71	-0.63
By age		
60–64 years	-0.35	-0.23
65–69 years	-0.33	-0.11
70–74 years	-0.49	-0.34
75–80 years	-0.91	-0.87
80–85 years	-1.44	-0.97

^a^ Expressed in percent point difference. All differences were summed, and weights were applied for the number of LASA-respondents in each group

^b^ We estimated 1-year mortality in LASA by dividing the percentage that died between subsequent measurement waves by three, since the interviews were held with 3-year intervals. Exceptions were the interval between wave E/2B (2001–2003) and wave F (2005–2006), which was on average 3.7 years, and the interval between wave H/3B (2011–2013) and wave I (2015–2016), which was on average 3.6 years. For each year (t), the percentage that died in each age group (X) between two successive waves in LASA was calculated as follows: number of deaths in age group X (at the time of death) between the two waves/number of deaths + number of survivors in age group X (at January 1 in year t, where t is the mid-year between two waves)

^c^ Mortality data were updated since the publication of last cohort update in 2019 [3], this explains the small discrepancies in sum differences between this table and the results previously published [3]

Averaged across all LASA waves, non-mortality attrition amounted to 5.5% and mortality attrition to 11.0% in each interval between waves. Thus, two-thirds of all attrition in LASA is due to mortality. We conducted additional analyses on the determinants of non-mortality attrition, considering that determinants of mortality have been widely studied. Across all waves, we examined socio-demographic characteristics and participation history of the respondents, defined as the interview mode at the wave prior to drop-out, across waves and cohorts. In order to account for interdependency of observations of individuals who participated in multiple waves, we used Generalized Estimating Equations [12]. We found that non-mortality attrition was not significantly associated with sex and age, but was higher at lower levels of education (Table 3). Furthermore, non-mortality attrition decreased across waves, but was increasingly higher in the more recent cohorts (cohorts 2 and 3). Interview mode at the previous wave appeared highly consequential; all modes other than a complete face-to-face interview raised the likelihood of non-mortality attrition, and this was particularly so for telephone interviews with the respondent.

**Table 3:** Determinants of non-mortality attrition, in particular the role of interview mode at the previous wave (Results are averaged across waves, using Generalized Estimating Equations (GEE) with *n* = 18165 observations)

	GEE Model^a^		
	OR	95% CI	p-value
Sex, female	0.92	0.81–1.04	0.178
Age in years	1.00	0.99–1.01	0.896
Education in years	0.94	0.92–0.96	< 0.001
Wave	0.94	0.90–0.99	0.010
Cohort 1 (ref.)	1.00		
Cohort 2	1.59	1.26–2.00	< 0.001
Cohort 3	3.68	2.68–5.06	< 0.001
Interview mode: Face-to-face complete (ref.)	1.00		
Face-to-face incomplete	3.49	2.79–4.36	< 0.001
Telephone self	6.69	5.66–7.90	< 0.001
Telephone proxy	4.33	3.17–5.90	< 0.001

OR = Odds ratio; 95% CI = 95% confidence interval

^a^Adjusted for sex, age, education, wave, cohort, and interview mode

## Data harmonization

Data from LASA are regularly being used for integrative studies, such as meta-analyses, international collaborations, and harmonization projects. Amongst others, in recent years, LASA data has been included in meta-analyses of the NCD Risk Factor Collaboration (NCD-RisC) [13] and the Netherlands Cohorts Consortium (NCC) [14]. LASA has also participated in research projects that involved retrospective data harmonization, such as the Comparison of Longitudinal European Studies on Aging (CLESA) project [15], the MINDMAP project on urban environments and mental well-being [16, 17], and the Netherlands Consortium of Dementia Cohorts (NCDC) [18, 19], and in projects involving prospective data harmonization, such as in the European Project on Osteoarthritis (EPOSA) [20].

## Ancillary studies

**Fig. 3:**
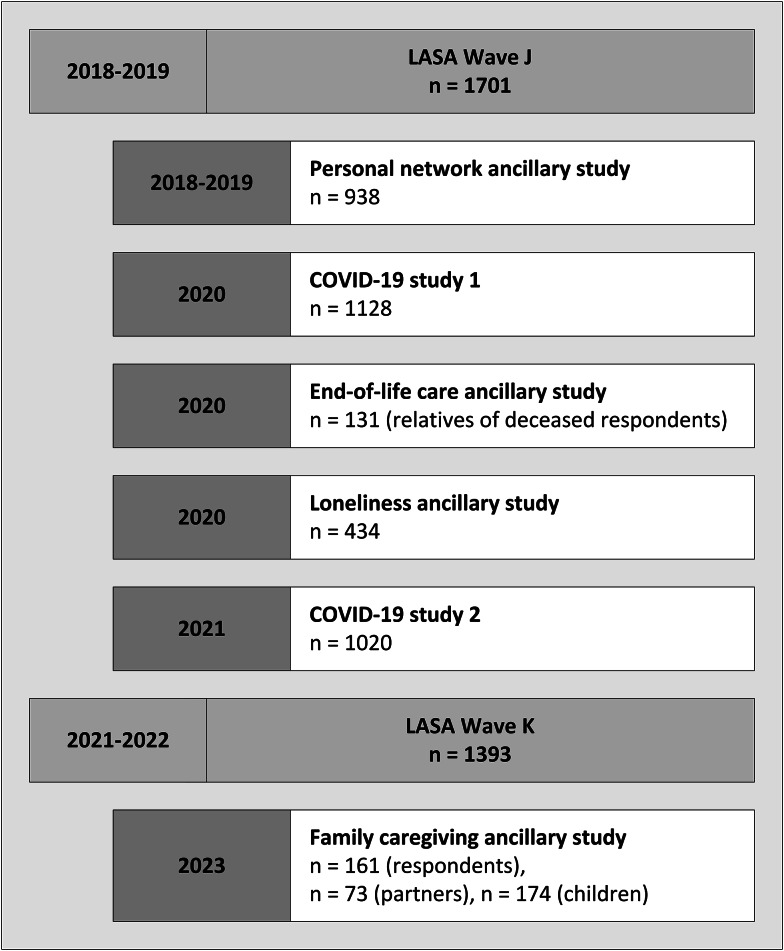
Timeline of LASA ancillary studies conducted since Wave J (2018–2019)

Over the years, several quantitative and qualitative ancillary studies among subsamples of LASA respondents have been performed to enrich the data of regular measurement waves. These ancillary studies were conducted to answer specific research questions or to gain a deeper understanding of a certain topic. For example, between the regular LASA measurements in 2015–2016 (wave I) and 2018–2019 (wave J), three additional nine-monthly measurements were performed among respondents aged 75 years and over (LASA 75 PLUS study). This increased density of measurements was performed to gain a better understanding of functioning among the oldest old (see [3] for more details). Figure 3 shows the ancillary studies that have been conducted since wave J. These ancillary studies have focused on personal network characteristics (2018–2019), COVID-19 (2020 and 2021), end-of-life (2020), loneliness (2020), and family caregiving (2023).

### Personal network ancillary study

We conducted an ancillary study to enrich LASA data about personal social networks in 2018–2019. The group of people with whom an individual interacts is a source of support in both daily routines and during stressful life events and affects health and well-being. It takes on special significance later in life, when personal health declines and the need for help increases. In many studies, including LASA, only functional relationship characteristics were examined, such as social support, strong relationships and positive ties with other people, whereas burdensome ties have usually been ignored. Although difficult relationships tend to be smaller in number than positive ties, their challenging nature makes them stressful for those involved. Their persistence poses a risk to mental health, for example, by evoking loneliness and feelings of disapproval from others, as well as to health and well-being in general. People may face structural and individual constraints that put pressure on them to interact socially with others, even if they sometimes find them demanding or difficult. It is plausible that these pressures increase as people age and it becomes more difficult to break or establish ties. Difficult relationships are not just a personal characteristic, but are often embedded in a network where more difficult relationships occur. Examining these requires a network approach. To do this, we collected additional data on characteristics of the relationships by following up to five individual network members from the main study.

At the end of the main interview of wave J (2018–2019), respondents were asked whether they would be interested in receiving an invitation to any of the upcoming ancillary studies. The vast majority of eligible respondents (*n* = 1193; 86%) agreed to receive an invitation to the Personal Network ancillary study. Approximately one month after the completion of the interview of the main study, respondents were mailed a personalized paper-and-pencil questionnaire that listed the personal network members selected for this study. Of the invited respondents, 79% returned the questionnaire (*n* = 938). These respondents did not differ meaningfully from non-respondents in sociodemographic and health variables, except for a slight overrepresentation of respondents who named children in their network in the main study (63% versus 54% among non-respondents).

The questionnaire started with a listing of the names of the five network members from the main study (first name and first letter of the last name) together with the relationship type to eliminate confusion about name duplicates, e.g., Jan B (cousin) and Jan B (neighbor). Relationships between network members were assessed by listing all possible pairwise combinations (e.g., Person A and Person B, Person A and Person C,…, Person D, and Person E). In addition, the questionnaire included additional questions on the respondent’s direct relationships with network members. Most notably information on relationship difficulty was asked, whether the respondents received criticism from the network member and whether the network member was too demanding. Questions were also asked about relationship quality and whether they meet or have contact by telephone or internet. Finally, general questions were asked about social integration in the neighborhood. After completing this study, the data was linked to the main study to construct complete egocentric networks.

### COVID-19 study

Just before the outbreak of the COVID-19 pandemic, LASA wave J (2018–2019) was completed. Because of the exceptional situation of the COVID-19 pandemic, which was expected to have serious consequences for the daily lives and functioning of older adults, two additional measurements were added in between wave J and wave K (2021–2022). This was done with a postal/online questionnaire that included measures specific for COVID-19 such as symptoms associated with SARS-CoV-2 infection at the time, changes in access to health care and social contacts, and changes in diet and physical activity. Additionally, the questionnaire included a selection of measures from regular LASA waves, covering multiple domains of functioning. Details on the questionnaire and the sample have been published previously [21]. In brief, of the 1701 respondents of the last pre-pandemic measurement (wave J), *n* = 1485 were selected for the first COVID-19 questionnaire, which was sent by postal mail in June 2020. Respondents were also given the opportunity to fill out the questionnaire online (digital questionnaire). Of the 1485 respondents approached, 1128 (76%) filled out the first COVID-19 questionnaire. In March 2021, a second questionnaire was sent to 1325 respondents, of which 1020 (69%) filled out the questionnaire [22].

Various studies using data from the LASA COVID-19 study have been published. One study was performed on the impact of the COVID-19 pandemic on nutrition and physical activity behavior [23]. About half of the LASA respondents reported a decrease in physical exercise and activity due to the pandemic. More than 20% reported changes in nutritional behavior, with overnutrition being reported more often than undernutrition [23]. In another study, a 35-item COVID-19 exposure index was developed to measure older adults’ cumulative direct and indirect exposure to the COVID-19 pandemic, such as COVID-19 infection of respondents and their close relatives, financial problems, restrictions in healthcare use, social contact, and physical activity [24]. Older adults with higher exposure to the COVID-19 pandemic showed worse functioning in the physical, mental and social domain [24].

Two studies on changes in medical care during the pandemic showed that one third of the LASA respondents reported cancelation of medical care during the first months of the pandemic, either initiated by the older person or by healthcare professionals [25]. In the second year of the pandemic fewer cancelations were reported [22]. Four studies were performed on mental health during the pandemic. In an early study it was found that loneliness, and especially emotional loneliness, increased during the pandemic in 2020 compared to 2019 [26]. A later study analyzing data from several ancillary studies up to 2024 showed that after the pandemic, levels of emotional and social loneliness had returned to pre-pandemic levels [27]. In the first year of the pandemic depressive and anxiety symptoms slightly increased [28]; in its second year anxiety and depressive symptoms stabilized [29]. Several factors were found to buffer the impact of COVID-19 related adversity on outcomes, for example COVID-19 vaccination buffered against COVID-19 exposure-induced anxiety and loneliness [29]. Other studies were performed on changes in giving and receiving care [30], personal development and meaning in life [31], and end-of-life treatment preferences during the pandemic [32].

### End-of-life care

The continued mortality decline during the past decades has shifted the age-at-death upwards. As more people die at older ages, the circumstances and experience of dying may change. Moreover, end-of-life care and access to care are likely to change in the wake of medical advances on the one hand and policy measures on the other hand. For example, an important change in the past decade on national policy level in the Netherlands is the emphasis on improving palliative care, which among others led to development of a quality framework palliative care. Therefore, LASA includes several questions about the end of life in the main study and in an ancillary study.

#### End-of-life care measured in the main study

Two, related, end-of-life topics are addressed: advance care planning (ACP) and medical end-of-life decisions. While these two main topics were assessed in most LASA waves, other end-of-life questions were added to one or two waves for specific projects at that moment. From wave D (1998–1999) onwards, questions were included about advance directives [33–35] and whether respondents had discussed their wishes for the end of life with others. As of wave I (2015–2016), ACP questions were added about specific treatments, for instance if people want to be admitted to a hospital or want to be resuscitated and with whom the respondent had discussed these wishes [32, 36]. Furthermore, as of wave G (2008–2009) also a more general question was asked about preferences: “When you think about the future, which do you prefer: 1) To live as long as possible, irrespective of health problems, or 2) To have a shorter life, if without major health problems” [37].

From wave B (1992–1993) onwards, questions were asked about LASA respondents’ opinion on requesting a doctor to end one’s life (euthanasia) or taking a suicide pill [38]. Moreover, in wave J (2018–2019) and wave K (2021–2022) a more general question was added under what circumstances LASA respondents would consider to use a suicide pill [39]. Furthermore, in waves F (2005-06), I (2015–2016), J, and K, questions were asked derived from the Paykel scale [40] about feelings and wishes about life and death [40–42].

#### The end-of-life care ancillary study

Since in the main LASA study respondents are interviewed with intervals of 3 or 4 years, we generally miss important information about the last months of life of LASA respondents. Therefore, an ancillary study among relatives of recently deceased LASA respondents about the last three months of life of the LASA respondent was initiated in 2000 [43], 2010 [44–46] and 2020, with an identical protocol. Table 4 shows information about the population of the ancillary end-of-life studies and the outcome measures included in the questionnaires. The ancillary studies have for instance shown that compared to 2000, in 2010, LASA respondents were significantly less likely to receive no care (12% vs. 39%) and more likely to receive formal home care (45% vs. 15%) in the last month of life, and that regardless of the study year, older people receiving informal home care were more likely to die in the hospital [45].

**Table 4:** Ancillary end-of-life study: data retrospectively provided by relatives of deceased respondents in LASA

	Sample 2000	Sample 2010	Sample 2020
Years of death of LASA-respondents	1996–1999	2005–2009	2017–2019
Proxies eligible^a^, n	342	311	215
Proxies traced, n	325	284	161
Proxies participated, n (% of proxies traced)	270 (83%)	167 (59%)	131 (81%)
Data collection mode	Face-to-face interview	Self-completion questionnaire	Self-completion questionnaire
Relationship of decedent to proxy			
Parent	55%	82%	66%
Partner	33%	10%	18%
Other relative	11%	8%	16%
Age range of decedents, years	60–91	60–100	60–104
Age at death, mean (SD)	79 (8)	81 (9)	82 (9)
Place of death			
Own home	35%	28%	31%
Hospital	33%	29%	14%
Care institution	32%	41%	50%
Other	0%	2%	5%
Measures	- Demographics of both proxy and decedent. - For the decedent: living arrangements, physical functioning, physical and mental symptoms, existing diseases, cause of death, need and receipt of care, treatment preferences, advance care planning, medical decisions, and quality of dying. Where relevant, these items were addressed regarding both 3 months and 3 days before death. - For treating physician (2000 only): diseases and medical end-of-life decisions. - For relatives (2020 only): family caregiving situation and caregiving tasks, burden, and grief.		

^a^ Inclusion criteria: the LASA-respondent had named a proxy to be contacted in case the study team would be unable to reach the respondent, and had not refused participation in the waves preceding their death

### Loneliness ancillary study

Loneliness is a problem common among older adults. It has serious health consequences, such as premature death [47, 48]. An ancillary study on loneliness was conducted in the fall of 2020 in collaboration with the Ministry of Health, Welfare and Sport to evaluate parts of a nationwide strategy to tackle loneliness. From the LASA respondents interviewed in 2018–2019 (*n* = 1591), those born in 1945 or earlier were selected for this ancillary study (*n* = 674). Data were obtained from 434 respondents. Reasons for non-response were death (*n* = 40); declined in advance (*n* = 65) or after being approached (*n* = 54); could not be found (*n* = 2); and ineligible due to frailty or for other reasons (*n* = 79). Data were collected between September 21, 2020, and January 18, 2021; the mean response date was November 8. Home interviews were planned, but because of the COVID-19 pandemic-related government measures, other methods were also used: 23% were interviewed at home, 44% were interviewed by telephone, 21% completed a written mail questionnaire, and 11% completed an online questionnaire. Their ages ranged from 74 to 102 years (mean = 81.4), 53% were women, and the majority (52%) lived with their partner in the household.

In the regular face-to-face and telephone interviews, loneliness is measured with the social-emotional loneliness scale, in the full version with 11 items [49] and in the short version with six items [50], respectively. Three direct questions about loneliness are also asked. In the ancillary 2020 data collection, existential loneliness, a sense of meaninglessness of life [51, 52], was additionally measured with seven items [26]. Five questions were asked about familiarity with and participation in activities that are organized by volunteer or welfare organizations to tackle loneliness, such as whether one had received home visits from someone from such organizations or the municipality, whether one was familiar with a free listening telephone service, and whether one was familiar with commercials broadcasted on television with the motto “Anyone can do something against loneliness.” Related to seven effective elements known against loneliness [53], seven pairs of two vignettes were presented about what contacts people have, and what happens in these contacts. Because place attachment, that is feelings of belonging to one’s home and environment, is assumed to counteract loneliness, a 13-item questionnaire about place attachment was included in the study [54]. Finally, questions were also asked about physical and cognitive functioning, falls, household and personal care obtained, and depressive symptoms.

The first results of this ancillary study showed that the three-factor model for emotional, social and existential loneliness fitted the data well [26]. So far, the data have been used in several studies on changes in loneliness around the COVID-19 pandemic, of which the results were described above in the section on the COVID-19 study [26, 27].

### Family caregiving ancillary study

In aging societies, the increased demand for informal care coincides with an expected scarcity of informal caregivers. One specific solution could be to share the care with others in a well-functioning family care network. To get more insight into family caregiving and a full understanding of what drives the use and provision of informal care in families, a LASA ancillary study was conducted: the FAMCARE project. This included an additional data collection among partners and children of older care recipients. The objective was to obtain information on whether and when care recipients, partners and adult children are willing and able to share the care, whether help from other caregivers complements or substitutes family care, and which features of the care network impact the wellbeing of caregivers and care recipients. The study builds on previous LASA studies on care networks [55] and its association with wellbeing of care recipients [56] by adding the perspective of family caregivers in a multi-actor study.

In the fall of 2023, we collected additional data from care recipients who participated in LASA wave K (2021–2022), their partner, and a maximum of three children. All respondents who met the following criteria were invited to participate: (1) living at home, (2) used at least one out of five types of care, (3) had at least one child, and (4) was cognitively able to participate indicated by a Mini-Mental State Examination (MMSE) score of at least 22, and (5) was eligible to participate in ancillary studies. A total of 425 respondents of wave K met these criteria and were approached. The partners (*n* = 75) and children (*n* = 294 from 168 respondents) were approached after approval by the respondent. If informed consent was obtained, data were collected in an interview or via a questionnaire. The respondent and their partner could choose between a face-to-face interview and a written questionnaire. Children always received an online questionnaire. The interview and questionnaires included various measures, such as health (physical, cognitive, mental health), use of care, relationship quality, personality characteristics, and various specific questions for the partner and the children on care tasks and caregiving experiences. Finally, in this ancillary study, 161 older adults (149 written questionnaires and 12 face-to-face interviews), 73 partners (72 written questionnaires and 1 face-to-face interview) and 174 children from 123 families (all online questionnaires) participated. The response rate of the LASA-respondents was rather low due to the fact that 232 of 425 (55%) approached respondents did not provide informed consent to approach their children. This may have resulted in some selection bias. Those respondents who participated (*n* = 161) were more often male, in better health and higher educated than respondents who did not participate (*n* = 264). The small sample size may require some adjustments of the analysis plan, such as the application of more simple statistical models than initially planned.

## Methods update

### Data linkage

Recently, data linkage between LASA and individual-level data available from Statistics Netherlands (CBS) has been established. This includes, for example, healthcare use and socioeconomic variables. Linkage is established through a probabilistic linkage, on the basis of information about postal code (6 digits), house number, date of birth, date of death (if applicable) and sex of the LASA respondent. Datasets that have been enriched by linkage with CBS individual-level data are accessible for specific research questions within a remote access environment that is hosted by CBS. For 4239 respondents (first cohort, *n* = 2796; second cohort, *n* = 760; third cohort, *n* = 683) additional information is currently available within this remote access environment. The main linked data available include: primary diagnoses reported in International Classification of Diseases 9 (ICD-9) or ICD-10 codes during hospital admission, i.e. clinical admissions, observations without an overnight stay and day admissions (available for the years 1995–2019), secondary diagnoses during hospital admission reported in ICD-10 (available for the years 2013–2019), yearly income (available for the years 2011–2022), yearly benefits (such as unemployment and illness benefits; available for the years 2011–2022), and work-related variables (such as part-time factor, weekly working hours and type of contract; available for the years 2010–2023).

### New and updated disease algorithms

As described previously [3], in LASA various data are combined into disease algorithms to provide a more accurate diagnosis in cases where a gold standard diagnosis is not available. The algorithms combine data such as self-reported symptoms and diseases, diagnoses reported by General Practitioners (GPs), and medication use. Below we provide an update of the available algorithms, and describe a new algorithm for identifying cases of Major Depressive Disorder (MDD).

Algorithms for seven cardiovascular diseases (CVD; angina pectoris, myocardial infarction, coronary artery disease, congestive heart failure, peripheral arterial disease, cardiac arrhythmia, and cerebrovascular accident), diabetes mellitus and osteoarthritis are now available for waves B (1992–1993) to I (2015–2016). These algorithms have been described previously [3] and can be found on the LASA website (www.lasa-vu.nl). Algorithms for metabolic syndrome and allostatic load have not been updated and are both available only at wave C. The Persistent Cognitive Decline (PCD) algorithm combines longitudinal data on cognitive decline obtained from respondent and proxies, data on dementia diagnosis from GP’s, interviewer observations, whether a respondent was living in a psychogeriatric ward, and data on causes-of-death into a single measure indicating the likelihood that the respondent had dementia at a specific measurement wave. The algorithm is now available from wave C (1995–1996) to wave I (2015–2016). At wave I, an additional *n* = 30 respondents (1.5%) were identified by the algorithm as having dementia, which makes a total of *n* = 356 dementia cases in the three LASA cohorts.

LASA includes extensive diagnostic interviews for depressive and anxiety disorders (i.e. the Diagnostic Interview Schedule (DIS [57]) and the CIDI [58]. However, these data may still give an incomplete picture of the prevalence and incidence of MDD in LASA. First, these interviews are only offered to respondents scoring ≥ 16 on the Center for Epidemiologic Studies– Depression scale (CES-D [59]) and/or ≥ 8 on the Hospital Anxiety and Depression Scale– Anxiety subscale (HADS-A [60]) during their general interview. Second, some respondents refuse to participate in the diagnostic interview. And third, we may miss episodes of depression occurring in-between waves. Therefore, similar to PCD, we recently developed a depression algorithm to optimize determination of the likelihood of MDD at each wave. The algorithm combines four indicators of depression for which data are available. In addition to the diagnostic interviews, these are CES-D scores ≥ 16, which have good sensitivity and specificity for MDD [61], use of anti-depressant medication, and GP data indicating presence of depressive disorder– with or without involvement of a medical specialist. The status of each of the four depression indicators could be: negative, positive, possible (meaning GP diagnosis ‘yes’ but without information on the year of diagnosis), missing, or not applicable (for example if no CIDI diagnosis was available because the respondent scored < 16 on the CES-D questionnaire). The algorithm distinguishes 48 possible combinations of these four indicators, which were assigned to one of five levels of likelihood of MDD: no, unlikely, possible, likely or very likely MDD.

Having a positive diagnosis based on the DIS/CIDI interview or based on GP records where a specialist was involved were considered as sufficient conditions for categorization as ‘very likely’ MDD, regardless of the status of the other indicators. Examples of ‘likely’ categorization are: diagnosis by a GP without specialist involvement and no other available data, and GP diagnosis without a year but positive on antidepressants use. The algorithm is currently available for waves B to I. Based on the algorithm, *n* = 550 LASA respondents (10.8%) had ‘very likely’ depression on at least one observed wave. This estimate seems plausible when comparing to the lifetime prevalence of MDD in other population-based studies (e.g. 9.8% in persons aged 65 or older in Kessler et al. [62], and 20.0% in persons aged 55 to 65 in de Graaf et al. [63]).

### Frailty

Frailty is a condition that involves the decline in multiple physiological systems, resulting in increased vulnerability to stressors [64]. Older adults living with frailty have an elevated risk for various adverse outcomes, such as falls, hospital admission, and mortality [64]. In the literature, there are two dominant operational definitions of frailty: the frailty phenotype [65] and the frailty index [66]. The frailty phenotype defines frailty as the presence of at least three out of five criteria: weight loss, exhaustion, slow gait speed, low grip strength, and low physical activity. The frailty index is a continuous score based on the accumulation of health deficits, with a higher number of deficits indicating higher frailty. Both frailty definitions have been operationalized with LASA data. From wave C onwards, algorithms for the frailty phenotype have been created. For LASA waves C to K, data files with frailty index scores have been established [67].

Various LASA studies have included one or both frailty instruments, such as on the predictors of mortality in old age [68–70], frailty trajectories [71, 72], historical trends in frailty and its association with mortality [7], the temporal association between frailty, social isolation and loneliness [73], adverse childhood circumstances as predictor of frailty [74], the phenotypic and genotypic association of grip strength with frailty [75], and the order in which frailty components emerge [76]. The frailty index was also used to compare frailty between older adults from Turkish and Moroccan origin and native Dutch, which showed large inequalities in frailty between immigrants and native Dutch, with higher frailty rates among immigrants [77]. An important question for clinical practice is whether frailty should be measured repeatedly or whether a single measurement is enough to predict adverse health outcomes. The work of Stolz et al., using LASA data on the frailty index, showed that changes in frailty are important to consider when predicting mortality in later life, because changes in frailty predicted mortality independently of frailty measured at just one time moment [78, 79].

### Intrinsic capacity

In 2015, the World Health Organization (WHO) introduced the concept of intrinsic capacity (IC), defined as a composite measure of an individual’s physical and mental capacities [80]. This multi-dimensional construct includes five domains: vitality, locomotion, sensory perception, cognition, and psychological well-being [81]. The WHO emphasizes that healthy aging involves more than just the absence of disease; it also includes the ongoing maintenance and enhancement of IC throughout the aging process. In LASA, a cross-sectional composite IC score was developed and validated using a formative approach. Koivunen et al. [82] utilized multiple regression to identify the most relevant indicators linked to the IC construct, employing 6-year functional decline as an outcome. The structural validity of the score was further assessed by evaluating whether the selected indicators represent all five domains of the IC construct as well as known group and criterion validity of the constructed summary score. The developed IC summary score demonstrated discriminative ability between age groups and according to health status and was shown to be associated with subsequent functional decline and mortality. This cross-sectional composite score is available for the first two LASA cohorts at measurement wave C (1995–1996) and F (2005–2006), respectively. The total IC and domain scores are available for 1908 individuals aged 57 to 88 years.

Furthermore, we developed longitudinal scores for IC and its domains within LASA, taking into account the latest insights from the WHO and other recent evidence [83]. This involves considering the inclusion of new indicators to fully capture IC. The longitudinal composite IC score, along with scores for its five domains, has recently become available [84]. It was found that IC scores declined with age and higher composite IC scores were associated with fewer functional limitations over time [84]. Researchers may now explore aging dynamics in IC over time, identify factors influencing healthy aging, and examine interaction effects between IC and environmental factors in their relation with healthy aging.

### Appetite and malnutrition

Poor appetite frequently occurs in older adults and is considered an important risk factor for the development of malnutrition [85]. In all LASA waves, information on appetite is available from the CES-D item ‘During the past week, I did not feel like eating, my appetite was poor’. This information is frequently used in research [86, 87]. Recently, additional, more specific assessments of appetite have been added to the LASA study. For example, the Nutrition and Food-related Behaviour ancillary study (2014–2015) includes the 4-item Simplified Nutritional Appetite Questionnaire (SNAQ) [88], one item on change in appetite during the past 4 weeks based on the Beck Depression Inventory II [89], and poor interoceptive awareness (IA) measured with two items of the 10-item IA-scale of the Revised Eating Disorder Inventory-II [90, 91]. These data have been used to investigate the general characteristics of older adults with poor appetite [92] as well as their dietary characteristics [93]. SNAQ has demonstrated predictive validity for various health outcomes, including weight loss [88], sarcopenia [94], and mortality [95]. A recent study validated different appetite measures, including SNAQ, against a gold standard in a test meal setting [96].

In 2017–2018, the MICrobiota, Malnutrition, and Appetite in Community-dwelling older adults (MicMAC) ancillary study was conducted among 359 LASA respondents aged 65 years older (for inclusion criteria, see the LASA website). Body composition, dietary intake, appetite and self-reported taste and smell problems were assessed. A sensory test battery was used to objectively determine gustatory function (sweet, sour, salty, bitter, and umami) and olfactory function (identification, discrimination, and threshold). Faecal samples and tongue swaps were collected to determine the microbiota through 16 S rRNA sequencing. Associations of poor appetite with taste and smell [97], oral microbiota [98], and gut microbial characteristics [99] have been reported.

Furthermore, the LASA 75 PLUS study, conducted between 2016 and 2019, includes five consecutive nine-monthly assessments of appetite using one-week diaries. Respondents were asked to record for 7 consecutive days their experience of appetite using the question ‘How do you rate your appetite today?’ with response options very poor, poor, moderate, good, and very good. One of the findings was that poor sleep, low mood (more strongly in females) and more severe pain (males only) were associated with poor appetite in older adults on a daily level both within and between persons [100]. More recently, in wave J (2018–2019) and wave K (2021–2022), the items ‘How would you rate your appetite of the past 7 days?’ (response options: good, fair, poor), and ‘Did you eat less than normal in the past 6 months, due to loss of appetite, gastrointestinal problems or problems chewing or swallowing?’ (response options: no, somewhat less, a lot less) were added. The latter item was included in order to be able to apply the Global Leadership Initiative on Malnutrition (GLIM) etiological criteria for diagnosing malnutrition [101]. Using these criteria and the SNAQ65 + criteria, the prevalence of malnutrition in Dutch community-dwelling older adults was recently determined, which was 5.4% based on SNAQ65 + and 7.1% based on GLIM, respectively [102].

### Information and communication technologies (ICT) data

Measuring the use of information and communication technologies (ICT) within a longitudinal study that has been conducted for more than 30 years presents a substantial challenge due to the rapid evolution of these technologies. In response to this, LASA ICT measures have been repeatedly adapted over successive waves from wave E (2001–2002) onwards. These measures encompass several domains: (1) use of computers, internet, and cell phones, and reasons for non-use; (2) functions of internet use, (3) online social interactions, and (4) receiving ICT-related support.

The adoption of ICT has shown remarkable growth: in 2001, 21% of our respondents used computers, and by 2021 this had gone up to 94%. Internet use has risen from 12 to 92% in the same period. In the first of these two decades, younger and higher educated respondents were the most likely to start using the internet [103]. At most LASA measurement waves, respondents were first asked a generic question on their use of computers, cell phones, and the internet, and this question has been updated several times to include new devices (e.g. smart phone, tablet). Follow-up questions were asked on how much time per week (wave E to wave I) or per day (waves J and K) they spend using computers and cell phones; non-users are asked about their reasons for non-use, including health reasons and lack of support (waves H, I, J and K). Two LASA studies have shown that internet use may have favorable outcomes. Berner et al. [104] found that internet use was associated with reduced cognitive decline and Holwerda et al. [29] found that it was associated with lower levels of depression during the COVID-19 pandemic.

LASA typically asks about purposes of internet use rather than about the use of specific applications. In wave E (2001–2002), respondents were asked about seven purposes, such as surfing for information and emailing. In wave H (2011–2012), this increased to nine, with new items on ordering medicine and groceries online, as well as visiting medical websites. In wave J (2018–2019), the list was expanded to 15 items, incorporating online healthcare and contact with medical professionals, recreational functions, and the use of government websites. Despite these alterations, many options remain available for longitudinal analyses of specific items. Two generic questions on online self-disclosure were included in waves E, F, and G. Social internet has been given considerable attention in LASA. Since wave H, the frequency of online engagement with family, friends, and neighbors has been documented. A recent LASA study indicated that such engagement contributes to the maintenance of social networks in older adulthood [105]. Finally, questions were asked about assistance with the use of computers, tablets, and smartphones from wave H onwards. Respondents who indicated they needed assistance received a follow-up question about whom they could ask to provide the support.

## Data Availability

LASA data are available for research. The LASA Steering Group has adopted a policy of sharing of data with interested researchers for specific research questions on aging-related issues. To obtain data, researchers need to submit an analysis proposal that is evaluated by an evaluation committee of LASA core experts, who evaluate proposals based on scientific quality and correspondence of the research goals with participants’ informed consent. Data are available for investigation under the condition that results of analyses will be made available to the research community, regardless of the results of the study. More information on data requests can be found on the LASA website: www.lasa-vu.nl. Forms to request use of blood to assess biomarkers are also available here.
